# Composition and Activity of the Non-canonical Gram-positive SecY2 Complex*[Fn FN2]

**DOI:** 10.1074/jbc.M116.729806

**Published:** 2016-08-22

**Authors:** Mikaila Bandara, Robin A. Corey, Remy Martin, J. Mark Skehel, Ariel J. Blocker, Howard F. Jenkinson, Ian Collinson

**Affiliations:** From the ‡ School of Oral and Dental Sciences, Lower Maudlin Street, Bristol BS1 2LY,; the § School of Biochemistry and; ¶ School of Cellular and Molecular Medicine, University of Bristol, University Walk, Bristol BS8 1TD, and; ‖ Biological Mass Spectrometry and Proteomics, Medical Research Council Laboratory of Molecular Biology, Francis Crick Avenue, Cambridge CB2 0QH, United Kingdom

**Keywords:** ATPase, complex, membrane, secretion, transport, SecY2, Streptococcus gordonii, accessory Sec system, translocon

## Abstract

The accessory Sec system in *Streptococcus gordonii* DL1 is a specialized export system that transports a large serine-rich repeat protein, Hsa, to the bacterial surface. The system is composed of core proteins SecA2 and SecY2 and accessory Sec proteins Asp1–Asp5. Similar to canonical SecYEG, SecY2 forms a channel for translocation of the Hsa adhesin across the cytoplasmic membrane. Accessory Sec proteins Asp4 and Asp5 have been suggested to work alongside SecY2 to form the translocon, similar to the associated SecY, SecE, and SecG of the canonical system (SecYEG). To test this theory, *S. gordonii secY2*, *asp4,* and *asp5* were co-expressed in *Escherichia coli*. The resultant complex was subsequently purified, and its composition was confirmed by mass spectrometry to be SecY2-Asp4-Asp5. Like SecYEG, the non-canonical complex activates the ATPase activity of the SecA motor (SecA2). This study also shows that Asp4 and Asp5 are necessary for optimal adhesion of *S. gordonii* to glycoproteins gp340 and fibronectin, known Hsa binding partners, as well as for early stage biofilm formation. This work opens new avenues for understanding the structure and function of the accessory Sec system.

## Introduction

*Streptococcus gordonii* is part of the viridans streptococci group along with *Streptococcus salivarius, Streptococcus mitis, Streptococcus mutans*, *Streptococcus oralis, Streptococcus parasanguinis* and *Streptococcus sanguinis*. Together, they form an important part of the microbiota of the human oral cavity (1). These organisms colonize tooth surfaces, developing complex microbial communities and forming biofilms, also known as dental plaque, which is strongly associated with dental caries and gum disease (2). *S. gordonii* can initiate bacterial colonization by creating surfaces for other bacteria to adhere to (3). If oral trauma occurs, *S. gordonii*, and other viridans streptococci, can enter the bloodstream, leading to bacterial binding of human platelets and formation of vegetations at cardiac sites. This gives rise to damage and dysfunction of the heart valves, characteristic of infective endocarditis (4). *S. gordonii* DL1 expresses a number of surface proteins linked with colonization and virulence, including antigen I/II proteins (SspA and SspB) (5), fibronectin-binding proteins (CshA and CshB) (6), and serine-rich repeat glycoprotein Hsa (7). Hsa is characterized as a sialic acid-binding adhesin and hemagglutinin that has been shown to mediate binding of *S. gordonii* to sialylated carbohydrate structures on human platelets and salivary glycoproteins (7, 8). Hsa, and homolog GspB, has also been shown to be involved in forming biofilms and oral colonization by *S. gordonii* (7–9). Most proteins expressed on the *S. gordonii* surface are transported by the general Sec pathway, but *S. gordonii* also contains a specialized export system seemingly dedicated to the transport of Hsa, known as the accessory Sec system (10). The core components of the accessory Sec system are SecA2 and SecY2 (homologs of general Sec proteins SecA and SecY, respectively (11)), along with three accessory Sec proteins: Asp1, Asp2, and Asp3 (12).

**FIGURE 1.**
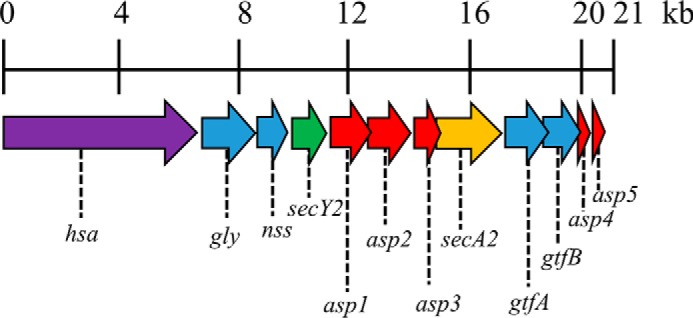
**Accessory *sec* locus of *S. gordonii* DL1.** Schematic representation of the ∼20.5-kb accessory *sec* locus (derived from the genome sequence of *S. gordonii* CH1: GenBank^TM^ accession number CP000725.1). Surface protein, *hsa* (SGO_0966); glycosyltransferases, *gly* (SGO_0968), *nss* (SGO_0969), *gtfA* (SGO_0975), and *gtfB* (SGO_0976); accessory secretion proteins, *asp1* (SGO_0971), *asp2* (SGO_0972), *asp3* (SGO_0973), *asp4* (SGO_0977), and *asp5* (SGO_0978); *secY2* (SGO_0970), *secA2* (SGO_0974).

Asp1–3 have been studied considerably in *S. gordonii* and have been shown to be essential for substrate export (12–14). Asp1–3 lack similar sequence homology to any known proteins, and due to their lack of signal peptides Asp1–Asp3 are expected to function intracellularly, where only Asp2 has a predicted transmembrane domain. Indeed, Asp1–3 have been shown to form a complex that is soluble and cytosolic but will also partially localize to the membrane when associated with SecA2 (15). The *secA2-secY2* locus encodes the serine-rich substrate Hsa along with core proteins, as mentioned above, for protein export and genes (*gtfA, gtfB, gly,* and *nss*) encoding glycosyltransferases necessary for glycosylation of Hsa (Fig. 1). Glycosylation is not required for transport but seems to influence protein stability and solubility. The glycosylation does however preclude export by the canonical Sec machinery and hence the requirement for an adapted machinery capable of glycoprotein secretion (16, 17).

The predicted membrane topology of *S. gordonii* SecY2 is almost identical to that of SecY; therefore, SecY2 is presumed to form a transmembrane channel to allow translocation of proteins across the cytoplasmic membrane (18). Disruption of *secY2* results in loss of substrate export similar to a *secA2* mutant, demonstrating that SecY2 is essential for a functional SecA2-SecY2 accessory Sec system (10). In the general Sec system, the translocon consists of SecY in association with small proteins SecE (14 kDa) and SecG (11 kDa) to form SecYEG. SecA associates with the SecYEG complex to drive translocation across the membrane (19, 20). Like SecY, SecE is essential for protein export, required stability, and for the integrity of the active protein channel (20–23). Although non-essential, SecG enhances translocation efficiency and becomes important if SecA function is disrupted (24–26). Some streptococcal species (*e.g. S. gordonii, Streptococcus pneumoniae,* and *Streptococcus agalactiae*) that possess the SecA2-SecY2 system also produce two additional small proteins known as Asp4 and Asp5 (Fig. 1) (27). Asp4 and Asp5 have similar sequence homologies to *Bacillus subtilis* SecE (52% similar) and SecG (55% similar), respectively, and have predicted transmembrane regions; therefore, it has been suggested that these proteins interact with SecY2 to form a translocon (18, 27, 28).

Transmembrane prediction for Asp4 and Asp5 indicates topologies with 1 and 2 transmembrane segments, respectively (supplemental Fig. S1). Sequence alignment of the predicted Asp4 and Asp5 transmembrane segments fits well with known transmembrane segments of SecE and SecG in other organisms (supplemental Fig. S2) (21, 29, 30). Additionally, Asp4 has a predicted amphipathic helical domain, a conserved structural feature observed in SecE (supplemental Fig. S1) (21, 29). Thus, the predicted topology and transmembrane segments of Asp4 and Asp5 appear to be evolutionarily conserved with SecE and SecG (supplemental Fig. S2). It has been suggested that Asp4 and Asp5 form a transmembrane complex with SecY2, but it has yet to be formally demonstrated.

We set out to test this hypothesis, through heterologous co-expression of codon-optimized *secY2*, *asp4*, and *asp5* in *E. coli* and affinity purification to determine whether these proteins interact with one another. We also provide evidence that Asp4 and Asp5, and hence the intact accessory complex, are required for optimal adhesion of *S. gordonii* to glycoproteins gp340^2^ and fibronectin, as well as for early stage biofilm formation.

## Results

### 

#### 

##### Purification of the Non-canonical Translocon Complex

**FIGURE 2.**
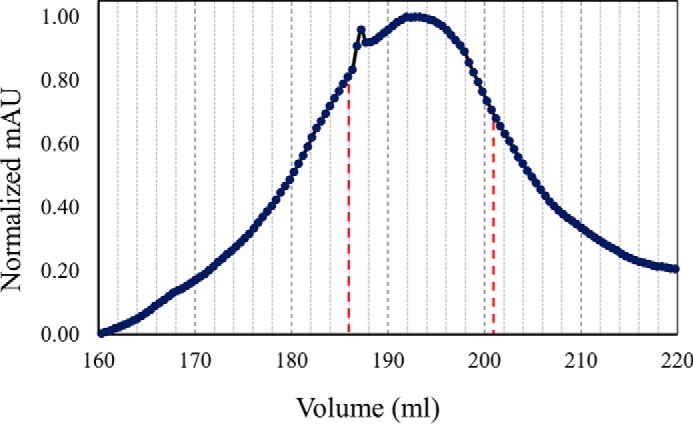
**Gel filtration chromatography of SecY2-Asp4-Asp5.** Trace from size exclusion chromatography of SecY2-Asp4-Asp5. Fractions volumes for SecY2-Asp4-Asp5 (186–201 ml) that were collected are represented by *red dashed lines*. The *column* was run in TSG_130_ buffer containing 0.02% DDM. *mAU*, milli-absorbance units.

**FIGURE 3.**
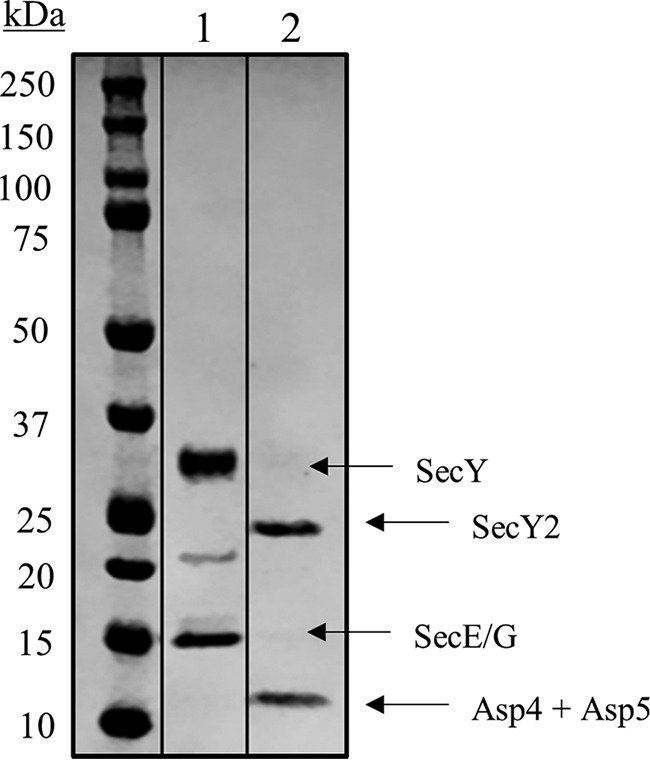
**SDS-PAGE analysis of purified SecY2-Asp4-Asp5 complex.** Coomassie Blue-stained gel of purified components. *Lane 1, E. coli* SecYEG (4 μg of protein), with bands for SecY and SecE/G indicated. Note that at ∼22 kDa, a SecY breakdown product is often present. *Lane 2, S. gordonii* SecY2-Asp4-Asp5 (4 μg protein), with bands at 25 and 10 kDa. These were subjected to LC-MS, where the 25-kDa band corresponded to SecY2, whereas Asp4 and Asp5 co-migrated at a molecular mass of 10 kDa.

**FIGURE 4.**
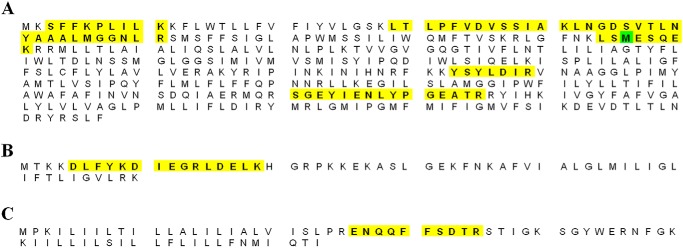
**Mass spectrometry peptides identified within the sequences of SecY2-Asp4-Asp5.** Isolated SecY2-Asp4-Asp5 samples were subjected to LC-MS analysis to identify the components present. The amino acid sequences of the proteins are shown above: *A,* SecY2; *B,* Asp4; and *C,* Asp5. *Yellow highlights* are the identified peptides within each protein sequence. *Green highlights* predicted post-translational modification.

SecY2-Asp4-Asp5 was purified by nickel affinity and gel filtration chromatography (Fig. 2). The purified complex was then subjected to SDS-PAGE analysis, alongside the *E. coli* canonical counterpart SecYEG (Fig. 3). The predicted molecular mass values of SecY2, Asp4, and Asp5 are ∼46, 7, and 8 kDa, respectively. The *E. coli* SecY (48 kDa) protein is known to migrate irregularly on SDS-PAGE running between 25 and 37 kDa (31). For this reason, the ∼25-kDa protein band in Fig. 3 was believed to be SecY2 and the ∼10-kDa band His-Asp4 and Asp5. To confirm their identities, each band was excised and subjected to liquid chromatography-mass spectrometry (LC-MS). LC-MS analysis positively identified the presence of SecY2, Asp4, and Asp5 with 18, 25, and 14% peptide coverages, respectively (Fig. 4). Other proteins identified by mass spectrometry are shown in supplemental Tables S1 and S2.

##### Purification of SecA2

**FIGURE 5.**
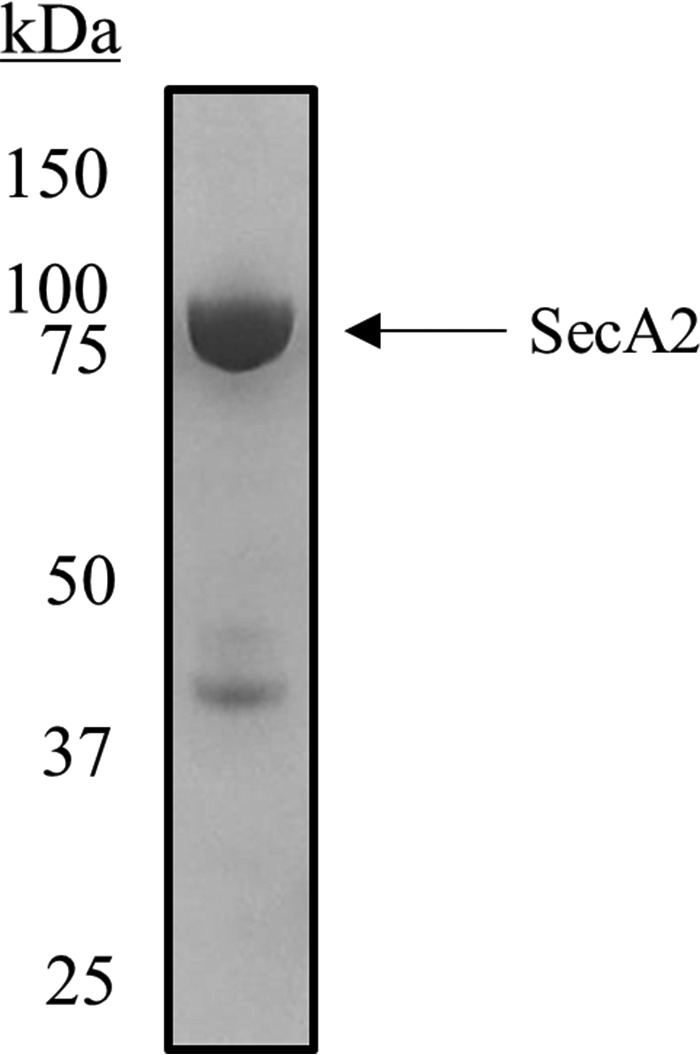
**SDS-PAGE analysis of purified SecA2.** Coomassie Blue-stained gel of purified *S. gordonii* SecA2 (2 μg of protein) with a band between 75 and 100 kDa (the predicted mass is 92 kDa). Protein bands between 37- and 50-kDa markers were presumed to be degradation products due to proteolysis.

Soluble SecA2 was purified from cytoplasmic extracts of overexpressing cells by nickel affinity, anion exchange, and gel filtration chromatography. The product was subjected to SDS-PAGE, and a band of the expected molecular mass (92 kDa) was visualized (Fig. 5). The lower molecular mass bands are degradation products, which were much more prevalent when protease inhibitors were omitted during the preparation.

##### ATPase Activity

The hydrolytic cycle of ATP of SecA drives protein translocation through the translocation channel of SecYEG (19, 32, 33); this ATPase activity is stimulated by SecYEG and further during the translocation process. ATPase assays were performed to see whether the activity of SecA2 could also be enhanced by SecY2-Asp4-Asp5.

**FIGURE 6.**
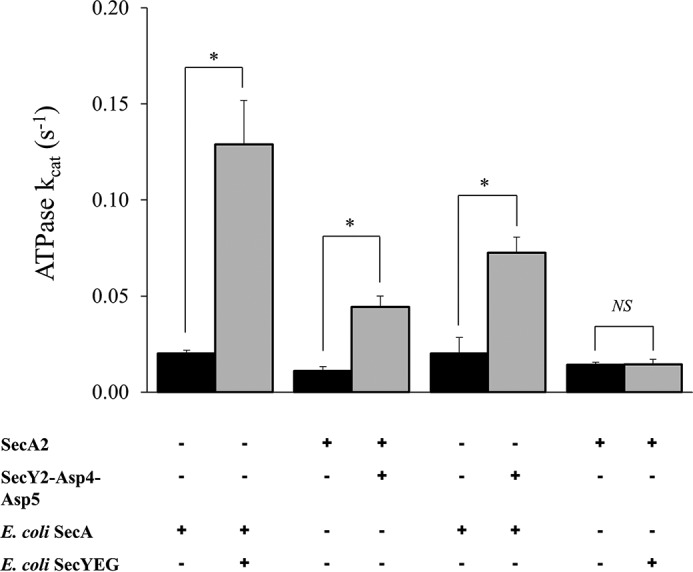
**ATPase activities of *S. gordonii* SecA2 with and without SecY2-Asp4-Asp5.** Steady-state ATPase activity of 0.3 μm *S. gordonii* SecA2 or *E. coli* SecA in the presence of 0.46 μm *S. gordonii* SecY2-Asp4-Asp5 or *E. coli* SecYEG, reconstituted in proteoliposomes. Statistical significance is indicated by an *asterisk* (*, *p* < 0.05, *t* test). No statistical significance (*NS*) is also indicated. *Error bars* are ± S.E. (*n* = 5).

Both the SecY2-Asp4-Asp5 and *E. coli* SecYEG complexes were reconstituted into PLS, providing near native conditions for the assay, and steady-state ATPase assays were performed. The ATPase activity of *S. gordonii* SecA2 was measured at ∼0.01 s^−1^ alone, whereas *E. coli* SecA was ∼0.02 s^−1^ (Fig. 6). Addition of SecY2-Asp4-Asp5 to SecA2 results in a 4-fold increase to about 0.044 s^−1^, whereas *E. coli* SecYEG has no discernible effect (Fig. 6). Addition of *E. coli* SecYEG to *E. coli* SecA results in a large stimulation of activity, to about 0.13 s^−1^, similar to previously reported data (34). Interestingly, *E. coli* SecA is also stimulated somewhat by SecY2-Asp4-Asp5 (0.072 s^−1^), implying a conserved mode of interaction between the different organisms and pathways (Fig. 6).

##### Model

**FIGURE 7.**
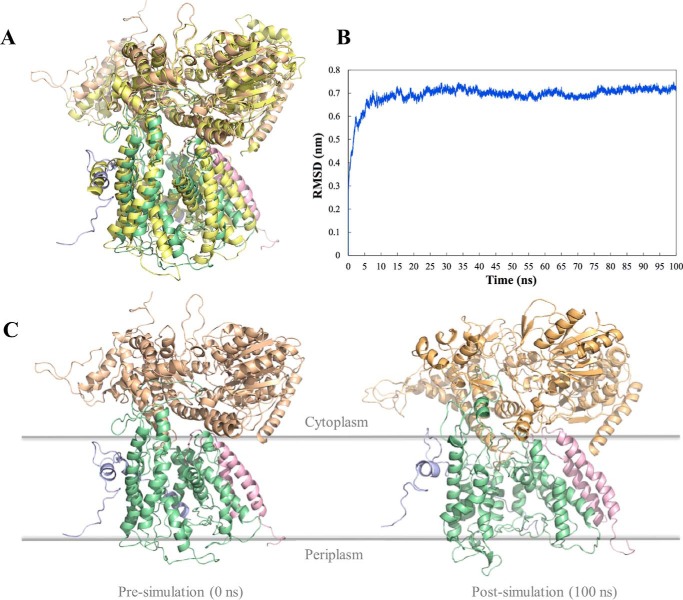
**Molecular dynamics simulation of SecY2-Asp4-Asp5-SecA2 model.** *A,* homology model of SecY2-Asp4-Asp5-SecA2 was created using Modeler and aligned to the crystal structure of SecYEG-SecA from *T. maritima* (shown in *yellow*), Protein Data Bank code 3DIN. *Green,* SecY2; *blue*, Asp4; *pink*, Asp5; *beige*, SecA2. r.m.s.d. of the alignment of the backbone atoms of the 2 structures is 0.95 Å. *B,* r.m.s.d. analysis of C-α atoms over the course of a 100-ns molecular dynamics simulation. The system stabilized within 10 ns, at an r.m.s.d. of ∼0.7 nm from its original position. *C,* pre- and post-simulation images of *S. gordonii* SecY2-Asp4-Asp5-SecA2 after 100 ns.

Sequence identity between the Gram-negative canonical SecYEG-SecA complex from *Thermotoga maritima*, of known structure (29), and the *S. gordonii* accessory Sec counterparts are ∼40, 24, 20, and 20% for SecA, SecY, SecE, SecG, respectively. This enabled the construction of a homology model of the non-canonical complex (Fig. 7*A*). To validate the structure, it was subjected to molecular dynamics simulation in a lipid bilayer environment. The overall structure was remarkably stable over the course of the simulation, equilibrating at a root mean squared deviation (r.m.s.d.) of 0.7 nm within 100 ns (Fig. 7, *B* and *C*).

Key structural features of SecY are apparent in the structural model and remain intact over the course of the simulation. The pore ring of SecY is highly conserved, and all but 1 residue remain close to their starting positions (supplemental Fig. S3). Three pairs of key residues flanking the SecY lateral gate are also seen in the SecY2 model (Thr-107/Tyr-250, Ile-110/Thr-253, and Ile-113/Ile-257). The respective distance between each of these pairs remains consistent during the simulation and stabilizes at an average distance of ∼2.3 Å after 50 ns, from a starting average distance of 1.9 Å.

##### Hsa Expression

**FIGURE 8.**
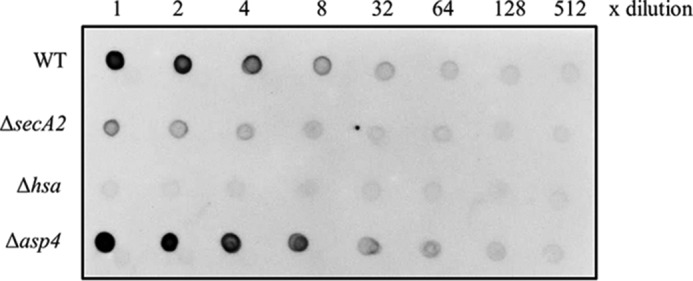
**WGA dot blot analysis of *S. gordonii* WT and mutants.** 2-Fold dilutions of intact cells of *S. gordonii* WT, Δ*secA2*, Δ*hsa,* and Δ*asp4* were dot blotted with WGA, which has primary sugar specificity to *N*-acetylglucosamine, a major component of Hsa.

Expression of SecA2-dependent protein Hsa on the cell surface of *S. gordonii* was determined by wheat germ agglutinin (WGA) dot blot. *S. gordonii* WT, Δ*secA2,* Δ*hsa,* and Δ*asp4* mutant strains were analyzed. Note that the *asp4* mutation was polar, therefore, the downstream *asp5* was not expressed (supplemental Fig. S4). WGA has primary sugar specificity to *N*-acetylglucosamine, a major glycosylation component of Hsa (35). Results confirmed the absence of surface expression of Hsa in the Δ*hsa* mutant and depleted surface expression of Hsa in the Δ*secA2* mutant (Fig. 8). In contrast, mutation of *asp4* did not appear to affect surface levels of Hsa. The WGA reaction with Hsa in the Δ*asp4* mutant was not significantly different from those shown in the WT (Fig. 8).

##### Biofilm Formation

**FIGURE 9.**
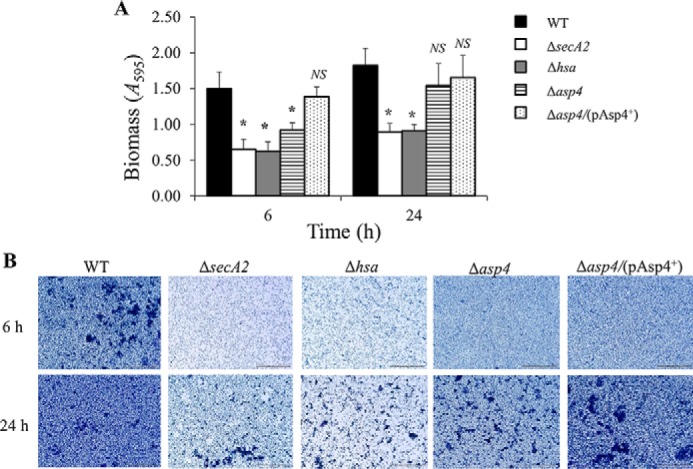
**Biofilm formation by *S. gordonii* WT, mutants as indicated, and *asp4* complemented strain.** *A, S. gordonii* monospecies biofilms were grown on saliva-coated coverslips for 6 or 24 h. Total biomass was quantified by crystal violet staining and measuring *A*_595_ following release of stain with acetic acid. Statistical significance to the wild type is indicated by an *asterisk* (*, *p* < 0.05, *t* test). No statistical significance to the wild type (*NS*) is also indicated. *Error bars* ± S.D. (*n* = 2). *B,* representative light micrographs of *S. gordonii* biofilms grown for 6 or 24 h and stained with crystal violet. *Scale bar,* 50 μm.

*S. gordonii* is an important primary colonizer of the oral biofilm and can recognize a large collection of salivary molecules such as gp340, proline-rich proteins, mucins, statherin, and α-amylase (36). To examine whether Asp4 is necessary for *S. gordonii* DL1 to form a robust biofilm, a monospecies biofilm assay was carried out. The assay entailed comparing *S. gordonii* WT, Δ*asp4* mutant, and *asp4* complemented strain together with Δ*hsa* and Δ*secA2* mutants as negative controls. A substantial reduction in biofilm formation was observed between the wild type and Δ*secA2* mutant, visually and quantitatively (Fig. 9).

Compared with the WT, biofilm formation for the Δ*secA2* mutant strain was decreased by 56 and 51% for the 6- and 24-h time points, respectively (Fig. 9*A*). A similar effect was obtained when examining biofilm formation for the Δ*hsa* mutant, a 58 and 50% decline for the respective 6-h and 24-h time points (Fig. 9*A*). When *asp4* was mutated, biofilm formation was significantly reduced by 38% at the 6-h time point. However, there was no significant change in biofilm formation between the WT and Δ*asp4* mutant after 24 h (Fig. 9). The *S. gordonii* Δ*asp4*/(pAsp4^+^) complemented strain produced biofilms comparable with the WT with no significant differences between the two (Fig. 9).

These results show that the biomass values for all *S. gordonii* strains in biofilms were slightly increased from 6 to 24 h (Fig. 9*A*). Although the Δ*asp4* mutant was reduced in biofilm formation during early stages of development, overall, the Δ*asp4* mutant and *asp4* complemented strain formed dense biofilms with clusters of micro-communities clearly visible, similar to the WT, whereas strains deficient in *secA2* and *hsa* were reduced in biofilm development (Fig. 9*B*).

##### Adhesion to Glycoproteins

**FIGURE 10.**
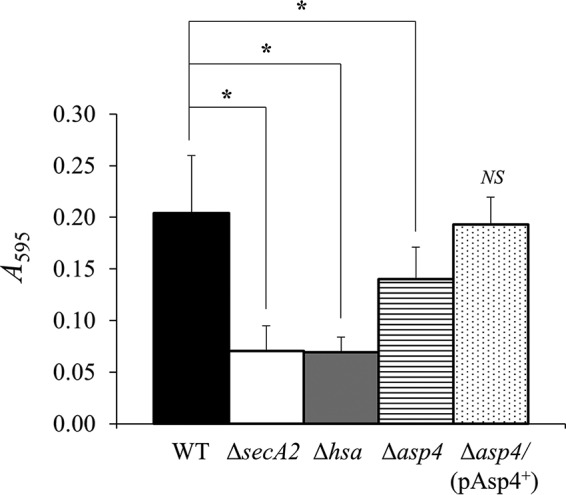
**gp340 binding by *S. gordonii* WT, mutants as indicated, and *asp4* complement strain.** gp340 (50 ng) was immobilized on the surface of MTP wells and incubated in the presence of bacteria. Bound bacterial cells were quantified by staining with crystal violet. Statistical significance to the wild type is indicated by an *asterisk* (*, *p* < 0.05, *t* test). No statistical significance to the wild type (*NS*) is also indicated. *Error bars* are ± S.D. (*n* = 5).

**FIGURE 11.**
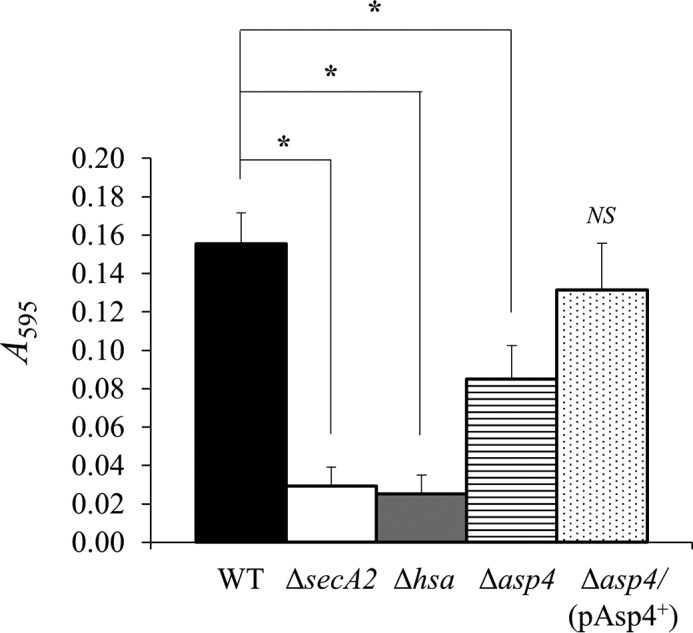
**Cellular fibronectin binding by *S. gordonii* WT, mutants as indicated, and *asp4* complement strain.** Cellular fibronectin (1 μg) was immobilized on the surface of MTP wells and incubated in the presence of bacteria. Bound bacterial cells were quantified by staining with crystal violet. Statistical significance to the wild type is indicated by an *asterisk* (*, *p* < 0.05, *t* test). No statistical significance to the wild type (*NS*) is also indicated. *Error bars* are ± S.D. (*n* = 4).

Hsa, secreted by the accessory Sec system, binds to sialic acid residues on a number of other glycoproteins such as salivary agglutinin gp340, bovine fetuin (blood proteins synthesized by the liver), and human fibronectin (5, 37, 38). Binding assays were carried out to identify whether mutating *asp4* affects adherence to glycoprotein gp340 and cellular fibronectin. For gp340 binding, both Δ*secA2* and Δ*hsa* mutants demonstrated reduced binding levels by ∼65% compared with the WT (Fig. 10). Mutation of *asp4* also resulted in reduced binding to gp340 with a significant decrease of ∼30% compared with the WT (Fig. 10). The *S. gordonii* Δ*asp4/*(pAsp4^+^) complemented strain exhibited similar levels of adhesion to gp340 as the WT (Fig. 10). For cellular fibronectin binding, both Δ*secA2* and Δ*hsa* mutants displayed an extensive 80% reduction in binding in comparison with the WT (Fig. 11). The Δ*asp4* mutant also exhibited reduced fibronectin binding, and there was a significant 50% decrease compared with the WT (Fig. 11). As expected, there was no significant difference in binding levels between the WT and *S. gordonii* Δ*asp4/*(pAsp4^+^) complemented strain (Fig. 11).

## Discussion

Asp4 and Asp5 are homologous to SecE and SecG, respectively (27). Both proteins have predicted transmembrane regions, and it has been proposed that Asp4 and Asp5 function as components of a membrane translocase together with SecY2 (SecY homolog) to form a translocation complex similar to the canonical SecYEG (13, 18, 39).

This study set out to test the hypothesis that Asp4 and Asp5 form a translocon with SecY2 (18, 28) by performing a biochemical pulldown assay on Asp4. Codon-optimized *secY2, asp4,* and *asp5* in *E. coli* were co-expressed and purified via the His tag on the N terminus of Asp4 to assess SecY2-Asp5-Asp4 interactions. *E. coli* SecYEG has been shown to purify as an intact complex when pulled down via His-tagged SecE (32, 40). The pulldown results, analyzed by LC-MS, validate that Asp4 and Asp5 localize to the cytoplasmic membrane in *E. coli*. This suggests that Asp4 and Asp5 likely form a membrane translocon with SecY2 in *S. gordonii*. Crucially, both SecY2 and Asp5 were detected thus confirming protein-protein interactions for SecY2, Asp4, and Asp5. The conformation of the equivalence of Asp4 and Asp5, respectively, with SecE and SecG suggests that the subunits fulfill a similar structural and functional role to those of the canonical protein channel complex.

Once the composition of the core non-canonical complex was confirmed, SecA2 was purified for analysis of functional interactions. SecY2-Asp4-Asp5 increased the ATPase activity of SecA2 indicating that collectively these proteins form an active complex. Interestingly, the SecA ATPase was also stimulated, although to a lesser degree, by addition of SecY2-Asp4-Asp5, but SecA2 activity was unaffected by addition of SecYEG. Thus, some directional promiscuity may occur, although this remains to be tested *in vivo*.

Some streptococcal strains that possess the SecA2-SecY2 system contain additional accessory Sec proteins Asp4 and Asp5 (27). *S. gordonii* and *S. pneumoniae* carry both Asp4 and Asp5, whereas *S. agalactiae* have only Asp4. Thus, the requirement of the additional proteins for export of substrates varies. Others have shown that Asp5 is required for GspB export in *S. gordonii* M99, and the loss of Asp4 significantly reduces, but does not abolish, GspB export (13). In this study, the *S. gordonii* Δ*asp4* mutant that was polar, thus affecting expression of *asp5*, exhibited similar surface expression of Hsa to the WT. However, the *asp4* mutation did inhibit biofilm formation at an early time point of 6 h. The mutation was possibly causing a lag for *S. gordonii* to produce a biofilm. Biofilm formation was not affected at 6- or 24-h time points when *asp4* and *asp5* were restored in the complemented strain as biomasses were comparable with the WT. The reduction in biomass for the Δ*asp4* mutant during the early development of biofilm could be due to Hsa not being exported as effectively to the bacterial surface as it normally would.

The Δ*asp4* mutant exhibited reduced binding to glycoprotein gp340 and to fibronectin, whereas the complemented *asp4* mutant was unaffected compared with WT. However, in this respect the Δ*asp4* mutant was much less affected compared with the Δ*secA2* mutant. This suggests that *asp4* and *asp5* are not essential for Hsa export. Nevertheless, they seem likely to play a role in Hsa functionality because of the mutational effects on *S. gordonii* binding to glycoproteins.

The stability of the canonical complex SecYEG complex is completely dependent on SecE (20–22) and to a lesser degree SecG. By analogy then, and given also their common structure (Fig. 7), the SecY2-Asp4-Asp5 complex would fail to assemble in the absence of Asp4. The reduced ability of the Δ*asp4* mutant for Hsa export and biofilm formation may be due to SecY complex or subunit promiscuity. Perhaps the activity *S. gordonii* SecYEG complex is sufficient for Hsa secretion and to support biofilm formation. (We were unable to test this possibility as the canonical *S. gordonii* SecYEG complex turned out to be very unstable). Alternatively, the *S. gordonii* SecE subunit might partially complement the missing Asp4 protein to support SecY2-SecE-Asp5 complex formation. The loss of SecA2 is more critical. So, this promiscuity is not a feature of the motor component. Therefore, the determinants of substrate specificity might be mostly defined by the motor component, rather than the channel. The SecY2 complex may serve simply to improve the efficiency of translocation of the SecA2 substrates during high demand. This might also explain why many organisms utilize a SecA2, without a SecY2 counterpart (28).

**FIGURE 12.**
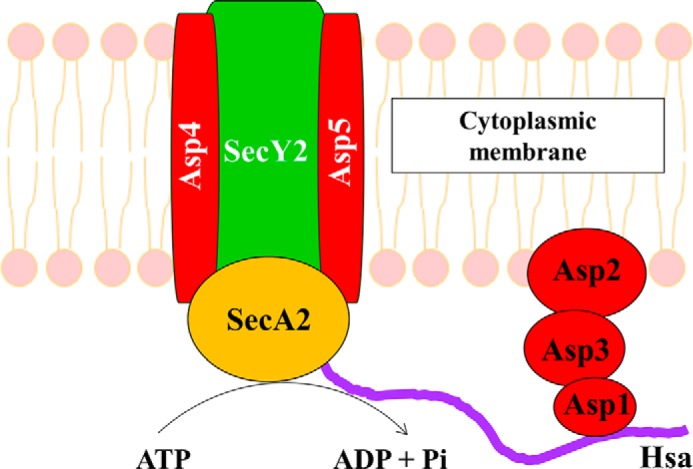
**Model of SecA2 bound to SecY2-Asp4-Asp5 translocon in *S. gordonii*.** SecY2, Asp4, and Asp5 form the cytoplasmic membrane translocon of the accessory Sec system, where SecA2 associates with the complex working as an ATPase to provide energy for Hsa substrate translocation.

We conclude that SecY2-Asp4-Asp5 most likely forms an active complex in *S. gordonii* DL1, where SecA2 can associate with the complex, thus powering protein export (Fig. 12). Upon association, the ATPase activity of SecA2 is stimulated *in vitro*, suggesting that the translocon performs protein translocation *in vivo*. However, the special adaptations of the accessory complex, for the export of a sub-set of unusual secretory proteins distinct from the broad spectrum of substrates of the canonical Sec system, have yet to be fully resolved.

## Experimental Procedures

### 

#### 

##### Bacterial Strains and Growth Conditions

The bacterial strains and plasmids used in this study are shown in Table 1. *Escherichia coli* strains were grown on LB agar (2.5% Luria Bertani (Difco) and 1.2% agar) aerobically at 37 °C. Single colonies were then transferred to 10 ml of LB broth (2.5% Luria Bertani) and incubated at 37 °C with shaking at 220 rpm. *E. coli* strains were grown in 2× YT broth (1.6% tryptone, 1% yeast extract, and 0.5% NaCl, pH 7.0). Media were supplemented with 100 μg ml^−1^ of ampicillin (Ap^R^). *S. gordonii* strains were grown on BHYN agar (3.7% brain heart infusion (LabM), 0.5% neo-peptone (Bacto), 0.5% yeast extract, and 1.2% agar). Single colonies were transferred to 10 ml of BHY broth (3.7% brain heart infusion and 0.5% yeast extract). Streptococcal strains were grown anaerobically (candle jar) at 37 °C. Media were supplemented with 100 μg ml^−1^ of spectinomycin (Sp^R^) or 2 μg ml^−1^ erythromycin (Em^R^) when necessary.

**TABLE 1:** **Strains and plasmids used in this study**

Strain or plasmid	Relevant characteristics	Ref. or source
**Strain**		
*S. gordonii* DL1	Wild type (Challis)	57
UB1744	Δ*secA2*::*aad9*; Sp^R^	37
UB2578	Δ*hsa*::*erm*; Em^R^	58
UB2637	Δ*asp4*::*aad9*; Sp^R^	This study
UB2700	Δ*asp4/* (pAsp4^+^); Sp^R^, Em^R^	This study
*E. coli* C43 (DE3)	F^−^ *ompT gal dcm hsdS_B_* (r_B_^−^ m_B_^−^)	Lucigen
UB2791	pBAD22-His*-asp4-secY2-asp5*; Ap^R^	This study
*E. coli* BL21 (DE3)	F^−^ *ompT gal dcm hsdS* (r_B_^−^m_B_^−^)	Novagen
UB2807	pBAD22-*secA2*-His; Ap^R^	This study

**Plasmids**		
pGEM®T	PCR product cloning vector; Ap^R^	Promega
pGEM®-T+Δ*asp4*::*aad9*	Containing upstream and downstream flanking regions of *asp4,* and *aad9* with transcriptional terminator	This study
pKS80	Lactococcal expression vector; Em^R^	43
pKS80-*gtfB-asp4-asp5*	Containing full-length *gtfB-asp4-asp5* genes	This study
pBAD22 (ATCC®87398^TM^)	Arabinose-inducible expression vector; Ap^R^	41
pBAD22-His*-asp4-secY2-asp5*	Containing full-length *asp4-secY2-asp5* genes. *asp4* contains a His_6_ tag on the N terminus	This study
pBAD22-*secA2*-His	Containing full-length *secA2* gene. *secA2* contains a His_6_ tag on the C terminus	This study

##### Generation of pBAD22-His-asp4-secY2-asp5

Gene synthesis of *asp4-secY2-asp5* with a His_6_ tag encoding sequence located on the N-terminal coding region of *asp4* was produced using GeneArt® (Life Technologies, Inc.). The gene sequences were codon-optimized for *E. coli,* and open reading frames were separated by *E. coli* spacer DNA containing ribosomal binding sites with restriction sites BamHI and KpnI incorporated at the beginning and end of the DNA sequence (total 1954 bp). His-*asp4-secY2-asp5* was digested with BamHI and KpnI restriction enzymes and ligated into BamHI- and KpnI-digested vector pBAD22 (4542 bp) (41). pBAD22 is a vector that has been used previously for cloning *E. coli secEYG*, where protein expression is under control of an arabinose-inducible promoter (40). The ligation mixtures were transformed into *E. coli* DH5α. The successful purified construct was confirmed by analytical digest and DNA sequencing prior to transformation into *E. coli* C43, a strain typically used to express membrane proteins, and designated UB2791.

##### Generation of pBAD22-secA2-His

Using chromosomal DNA of *S. gordonii* DL1 as a template, the *secA2* gene sequence was amplified by PCR using Phusion® high fidelity kit (New England Biolabs) and primer pair SecA2.pBAD22.F/SecA2.pBAD22.R (Table 2). The forward primer was designed with a 15-bp overhang (CAGGAGGAATTCACC) homologous to the sequence within vector pBAD22. The reverse primer was also designed with a 15-bp overhang (GGTACCAAATTCCAG) homologous to the sequence within vector pBAD22 with the addition of sequence CACCATCACCATCACCATTAG encoding a His_6_ tag and stop codon onto the C-terminal coding region of the gene. The whole length of purified vector pBAD22 was amplified by PCR using primer pair pBAD22.Fx/pBAD22.Rx (Table 2). Using the In-Fusion® HD cloning kit (Clontech), *secA2* was ligated into pBAD22 and transformed into *E. coli* DH5α. The successful purified construct was confirmed by analytical digest and DNA sequencing prior to transformation into *E. coli* BL21 and designated UB2807.

**TABLE 2:** **Primers used in this study**

Strain	Name	Sequence 5′ → 3′*^a,b^*	Target
UB2637	MBF9	GAAGGCTTAGAGGTCTTGG	Flank-*asp4*
	MBR9	**CAAAATCATCAAGC**GGATCCCTAACCGTCCCTCAATATCC	Flank-*asp4*
	MBF10	**TGAGGGACGGTTAG**GGATCCGCTTGATGATTTTGATTGGC	*asp4*-flank
	MBR10	GTTTCTTTCCCAATAACCAG	*asp4*-flank
	Asp5RT.F	CTGACTATATTGCTTGCTC	
	Asp5RT.R	GAATCATATTGAATAGGAGG	
UB2700	GtfB.F	GCGGGATCCAAATGATTCAGCTCTTTGATTATTACAATCAGG	*gtfb-asp4-asp5*
	Asp5.R	GCGCTGCAGCTAGATTGTTTGAATCATATTGAATAGGAGG	*gtfb-asp4-asp5*
UB2807	pBAD22.Fx	GGTACCAAATTCCAGAAAAGAG	pBAD22
	pBAD22.Rx	GGTGAATTCCTCCTGCTAGCCC	pBAD22
	SecA2.pBAD22.F	**CAGGAGGAATTCACC**ATGGTTAAAAACTTTTTTCATATTC	*secA2*-His
	SecA2.pBAD22.R	**CTGGAATTTGGTACCCTAATGGTGATGGTGATGGTG**TGGGAAGTACATTACGACCTC	*secA2*-His

*^a^* Restriction sites are underlined.

*^b^* Overhangs are in boldface.

##### Generation of the S. gordonii Δasp4 Mutant

Mutation of *asp4* in *S. gordonii* DL1 was achieved by allelic exchange with spectinomycin cassette *aad9*. Using chromosomal DNA of *S. gordonii* DL1, upstream (500 bp) and downstream (198 bp) flanking regions of *asp4* were amplified by PCR with primer pairs MBF9/MBR9 and MBF10/MBR10 (Table 2), respectively, using Expand Long Template PCR system (Roche Applied Science). Upstream and downstream flanking regions were ligated by PCR, generating an amplimer (*asp4*flank) with a central BamHI site that was cloned into pGEM-T in *E. coli* JM109. Using primer pair BamSpecF/BamSpecR, the spectinomycin resistance cassette *aad9* (1015 bp), with its own promoter and transcription terminator, was amplified from pFW5 (42) with terminal BamHI sites and cloned into the unique BamHI site within the vector pGEM-T-*asp4* flank. The resulting construct, pGEM-T-Δ*asp4*::*aad9* (1725 bp) was confirmed by restriction digest (SacII/SpeI), gel-extracted, and transformed into *S. gordonii* DL1 with selection for 100 μg ml^−1^ spectinomycin resistance. The successful transformant was identified by PCR screening and DNA sequencing. The *S. gordonii* Δ*asp4* mutant was designated UB2637.

##### RNA Extraction and RT-PCR

RNA was extracted from overnight streptococcal cultures using RNAprotect® bacteria reagent (Qiagen), and cells were disrupted with 15 mg ml^−1^ lysozyme and 100 units of mutanolysin before using RNeasy® mini kit (Qiagen) according to the manufacturer's instructions. To remove any remaining DNA from the RNA sample, a DNase digest was carried out. DNase reaction mixtures consisted of the entire RNA sample, 1× RQ1 DNase I buffer (Promega), 10 units of RQ1 RNase-Free DNase (Promega) in a total volume of 100 μl with RNase-free H_2_O. Reactions were incubated for 2 h at 37 °C. RNA clean-up was performed using RNeasy® mini kit. For synthesis of cDNA from the extracted RNA, an iScript^TM^ cDNA synthesis kit (Bio-Rad) was used according to the manufacturer's instructions. PCR was performed for each cDNA sample using primer pair Asp5RT.F/Asp5.RT.R (Table 2) to check RNA expression of *asp5.*

##### Complementation of the asp4 Mutant

Complementation of *asp4* in *S. gordonii* Δ*asp4* was generated using a simple cloning strategy. The mutation within *asp4* was polar as RT-PCR results demonstrated that *asp5* was not being expressed (supplemental Fig. S4). In the *S. gordonii* DL1 accessory *sec* locus, upstream gene *gtfB* overlaps *asp4*; therefore, it was decided to clone all three genes (*gtfB-asp4-asp5*) into vector pKS80 (43) to complement *asp4* and *asp5*. It must be noted that the complemented strain would also have two copies of *gtfB* as a consequence of cloning this along with *asp4* and *asp5*. Using primer pair GtfB.F/Asp5.R (Table 2), *gtfB-asp4-asp5* (1751 bp) was amplified with a BamHI site 5′ extension on GtfB.F and a PstI site 5′ extension on Asp5.R. Both pKS80 (5242 bp) and *gtfB-asp4-asp5* (1765 bp) were digested with BamHI and PstI restriction enzymes before ligation and transformation into *S. gordonii* Δ*asp4.* Successful transformants were selected for 2 μg ml^−1^ erythromycin resistance and 100 μg ml^−1^ spectinomycin resistance and confirmed by sequencing. The complemented *S. gordonii* Δ*asp4*/(pAsp4^+^) strain was designated UB2700.

##### Expression of SecY2-Asp4-Asp5 and SecA2

Overnight cultures of UB2791 or UB2807 were inoculated with a 1:10 dilution in shake flasks containing 2× YT broth with 100 μg ml^−1^ ampicillin (37 °C, 220 rpm). Cultures were induced at an *A*_600_ = 0.6 with 0.1% (w/v) arabinose for 3 h. Cells were harvested by centrifugation at 4800 rpm for 20 min at 4 °C (Sorvall Evolution RC). Pellets were stored at −80 °C.

##### Preparation and Purification of SecY2-Asp4-Asp5

Pellets were resuspended and homogenized in TSG_130_ buffer (20 mm Tris-HCl, pH 8.0, 130 mm NaCl, 10% (v/v) glycerol) and cells lysed on a cell disruptor twice at 25,000 p.s.i. (Constant Systems Ltd.). Membranes were separated from the cell lysate by centrifugation at 38,000 rpm for 45 min at 4 °C (Beckman Optima L-100-XP, Ti45 rotor). The collected membrane pellets were solubilized in TSG_130_ buffer containing 1% (w/v) *n*-dodecyl β-d-maltoside (DDM) for 1 h at 4 °C. Following DDM solubilization, the insoluble material was removed by centrifugation (38,000 rpm, 45 min, 4 °C). The DDM-soluble supernatant was retained on ice in preparation for purification. The detergent-solubilized protein preparation was applied to a chelating Ni^2+^-Sepharose fast flow column (GE Healthcare) equilibrated with TSG_130_ buffer containing 0.1% DDM. Following loading of the sample, the column was washed with TSG_130_ buffer containing 0.1% DDM and 30 mm imidazole, to wash any unbound or weakly bound protein through the column. Elution was performed with the same buffer containing 330 mm imidazole, collecting 1–5-ml fractions. Peak fractions were pooled and loaded onto a Superdex 200 26/60 gel filtration column (GE Healthcare) equilibrated in TSG_130_ buffer containing 0.02% DDM. Elution was performed with the same buffer, collecting 1–5-ml fractions. Pooled peak fractions were concentrated using a 10-kDa molecular mass cutoff centrifugation filter (Millipore), aliquoted, flash-frozen in liquid N_2_, and stored at −80 °C. Note that *E. coli* SecYEG was also expressed and purified as described previously (40).

##### Preparation and Purification of SecA2

Pellets were resuspended in TKM buffer (20 mm Tris-HCl, pH 8.0, 50 mm KCl, 2 mm MgCl_2_) and supplemented with protease inhibitor mixture-EDTA-free tablets (Roche Applied Science) and 0.6 mm PMSF to inhibit proteolysis. Cells were broken on a cell disrupter twice at 25,000 p.s.i., and cytoplasmic proteins were separated from the cell lysate by centrifugation at 38,000 rpm for 45 min at 4 °C. The supernatant containing cytoplasmic membranes was retained on ice in preparation for protein purification. The cytoplasmic protein preparation was applied to a chelating Ni^2+^-Sepharose fast flow column (GE Healthcare) equilibrated with TKM buffer. Following loading of the sample, the column was washed with TKM buffer and 30 mm imidazole to wash any unbound or weakly bound protein through the column. Elution was performed with the same buffer containing 330 mm imidazole, collecting 5-ml fractions. Peak fractions were pooled, and 1 mm dithiothreitol (DTT) was added. If fractions were left overnight, the imidazole was removed by dialysis using a 6000–8000-dalton porous membrane (Spectrum Labs). Pooled fractions were loaded onto a Q-Sepharose anion exchange column (GE Healthcare) equilibrated with TKM buffer containing 1 mm DTT. Following loading of the sample, the column was washed with TKM buffer containing 1 mm DTT to wash positively charged proteins through the column. Elution was performed with the same buffer containing 1 m KCl to elute strongly bound negatively charged proteins, collecting 5-ml fractions. Peak fractions were pooled and loaded onto a Superdex 200 26/60 gel filtration column (GE Healthcare) equilibrated in TKM buffer containing 1 mm DTT. Elution was performed with the same buffer, collecting 5-ml fractions. Pooled peak fractions were concentrated using a 30-kDa molecular mass cutoff centrifugation filter (Millipore), aliquoted, flash-frozen in liquid N_2_, and stored in −80 °C. Note that *E. coli* SecA was also expressed and purified as described previously (44).

##### Mass Spectrometry

Polyacrylamide gel slices (1–2 mm) containing the purified proteins were prepared for mass spectrometric analysis by manual *in situ* enzymatic digestion. Briefly, the excised protein gel pieces were placed in a well of a 96-well microtiter plate and destained with 50% v/v acetonitrile and 50 mm ammonium bicarbonate, reduced with 10 mm DTT, and alkylated with 55 mm iodoacetamide. After alkylation, proteins were digested with 6 ng μl^−1^ Trypsin (Promega, UK) overnight at 37 °C. The resulting peptides were extracted in 2% v/v formic acid, 2% v/v acetonitrile. The digest was analyzed by nano-scale capillary LC-MS/MS using an Ultimate U3000 HPLC (ThermoScientific Dionex, San Jose, CA) to deliver a flow of ∼300 nl min^−1^. A C18 Acclaim PepMap100 5 μm, 100 μm × 20 mm nanoViper (ThermoScientific Dionex), trapped the peptides prior to separation on a C18 Acclaim PepMap100 3 μm, 75 μm × 150 mm nanoViper (ThermoScientific Dionex). Peptides were eluted with a gradient of acetonitrile. The analytical column outlet was directly interfaced via a modified nano-flow electrospray ionization source, with a hybrid dual pressure linear ion trap mass spectrometer (Orbitrap Velos, ThermoScientific). Data-dependent analysis was carried out, using a resolution of 30,000 for the full MS spectrum, followed by 10 MS/MS spectra in the linear ion trap. MS spectra were collected over an *m*/*z* range of 300–2000. MS/MS scans were collected using a threshold energy of 35 for collision-induced dissociation. LC-MS/MS data were then searched against a protein database (UniProt KB) using the Mascot search engine program (Matrix Science, UK) (45). Database search parameters were set with a precursor tolerance of 5 ppm and a fragment ion mass tolerance of 0.8 Da. Two missed enzyme cleavages were allowed, and variable modifications for oxidized methionine, carbamidomethyl cysteine, pyroglutamic acid, phosphorylated serine, threonine, and tyrosine were included. MS/MS data were validated using the Scaffold program (Proteome Software Inc.) (46). All data were additionally interrogated manually.

##### ATPase Activity Assays

Purified translocons were reconstituted into PLS with 2-ml reactions containing 1.65 μm translocon protein, 5.9 mg of *E. coli* total polar lipid extract, and 300 g of BioBeads (Bio Rad). Reactions were dialyzed for 16 h in TKM buffer, and 75 mg ml^−1^ in BioBeads. Samples were then centrifuged at 80,000 rpm for 25 min at 4 °C (Beckman Coulter Optima, TLA 100.3 rotor) and resuspended in a total volume of 727 μl of TKM (to give a total concentration of 0.6 μm) and were aliquoted into 30 μl of flash-frozen in liquid N_2_ and stored at −80 °C. Steady-state ATPase measurements were carried out and monitored at 25 °C using a Lambda 25 spectrophotometer (PerkinElmer Life Sciences). ATPase activities were assayed in TKM buffer containing 0.2 mm NADH, 2 mm phosphoenolpyruvate, 1 unit of lactate dehydrogenase, and 1.4 units of pyruvate kinase in 100-μl cuvettes. Reactions were initiated by addition of 1 mm ATP and 0.3 μm *E. coli* SecA or *S. gordonii* SecA2. The change in absorbance was monitored at 340 nm for 20 min. To test translocation activity, 0.46 μm *E. coli* SecYEG or *S. gordonii* Asp4-SecY2-Asp5 PLS were added, and the absorbance was monitored for a further 20 min. The data for ATPase reactions were analyzed using Prism (GraphPad) and plotted on Excel (Microsoft®) using gradient slopes generated by the UVwinlab software (PerkinElmer Life Sciences). Values given represent the mean of five independent experiments.

##### Molecular Dynamics

A SecY2-Asp4-Asp5-SecA2 homology model for molecular dynamic simulations was built from the respective *S. gordonii* sequences, using Modeler (47). The crystal structure of the *T. maritima* SecYEG-SecA complex (Protein Data Bank code 3DIN) was used as a template (29), and bound nucleotide in SecA was removed. All simulations were performed using Gromacs 5.0.4 (48) and the GROMOS96 53a6 force field (49) with Berger lipid parameters (50). The homology model was inserted into an equilibrated 512 1-palmitoyl-2-oleoyl-*sn*-glycero-3-phosphocholine lipid bilayer (51) using g_membed (52). The defined simulation box was solvated with explicit SPC water molecules. Na^+^ and Cl^−^ ions were added to a total concentration of 150 mm and a neutral net charge on the system. The solvated system was energy-minimized using steepest descent minimization, for 50,000 steps or until maximum force <1000 kJ mol^−1^ nm^−1^. The system was subjected to a 100-ps NVT equilibration at 323 K using the stochastic velocity-rescaling thermostat. A subsequent 1-ns NPT ensemble equilibration at 323 K utilized the Nose-Hoover thermostat and semi-isotropic Parinello-Rahman pressure coupling. Production molecular dynamic simulations were run on Phase 3 of Blue Crystal, the University of Bristol's High Performance Computer. Simulations were run for 100 ns with 2-fs integration steps.

##### WGA Whole Cell Dot Blot

Bacteria were grown for 16 h, harvested by centrifugation (5000 × *g*, 7 min), and adjusted to *A*_600_ = 1.0 using fresh medium. Overnight culture (2 μl) was spotted directly onto a nitrocellulose membrane with 2-fold dilutions and dried for 10 min. Dot blot was performed with WGA as described previously (5).

##### Biofilm Assay

Unstimulated whole saliva was prepared as described previously (53). Sterile coverslips (19 mm diameter) were placed in each well of a 12-well polystyrene tissue culture plate (Greiner Bio-One), and 0.5 ml of 10% saliva was added. Plates were incubated at 4 °C overnight. Bacterial cultures were grown for 16 h at 37 °C and equilibrated to *A*_600_ = 0.1 with fresh broth. Saliva was removed from coverslips, and portions (0.5 ml) of culture were added to wells containing saliva-coated coverslips in quadruplicate for each strain. Biofilms were grown for 6 or 24 h anaerobically at 37 °C. Media were removed, and coverslips were rinsed in PBS. Biofilms were stained with 0.5% crystal violet (1 ml) for 15 min, washed with distilled H_2_O until excess stain was removed, and air-dried. For visualization of biofilms, coverslips were inverted and mounted onto microscope slides and viewed on a light microscope (Leica) with attached color view camera and images captured using CellD imaging software (Olympus Soft Imaging Solutions). For biomass quantification of biofilms, crystal violet was dissolved in 10% (v/v) acetic acid for 15 min, and 100-μl portions transferred to a microtiter plate (MTP). Absorbance at 595 nm (*A*_595_) was then measured (54) on an iMark^TM^ MTP reader (Bio-Rad). All studies were performed in triplicate, and mean biomass levels were calculated from two independent experiments.

##### Adhesion Assays

gp340 was prepared from parotid saliva samples pooled from multiple donors using a multistep procedure, including adsorption onto *S. mutans* as described previously (55). gp340 was diluted in coating buffer, and 50 ng of substrate was added per well to an Immulon 2 HB 96-well plate (Thermo-Scientific) at 4 °C for 17 h. Purified cellular fibronectin from human foreskin fibroblasts (Sigma) was diluted in coating buffer, and 1 μg of substrate was added per well to an Immulon 2 HB 96-well plate at 4 °C for 17 h. Adherence of *S. gordonii* cells to immobilized gp340 or fibronectin was performed by crystal violet assay as described previously (56). All studies were performed in triplicate, and values given represent the mean of 4–5 independent experiments.

## Author Contributions

M. B., A. J. B., H. F. J., and I. C. designed the research. M. B., R. A. C., R. M., and J. M. S. performed the experiments, analyzed the data, and prepared the figures. M. B., R. A. C., R. M., J. M. S., A. J. B., H. F. J., and I. C. wrote the manuscript.

## Supplementary Material

Supplemental Data

## Footnotes

2 The abbreviations used are:

gp340: glycoprotein-340
WGA: wheat germ agglutinin
PLS: proteoliposome
r.m.s.d.: root mean square deviation
DDM: *n*-dodecyl β-d-maltoside.
