# Supplementary material for: Composition and Activity of the Non-canonical Gram-positive SecY2 Complex
Source: J Biol Chem. 2016 Aug 22;291(41):21474–84. doi: 10.1074/jbc.M116.729806 (PMC5076819; doi:10.1074/jbc.M116.729806)
Supplement: Supplemental Data [file supp_291_41_21474__index.html]

Composition and activity of the non-canonical Gram-positive SecY2 complex — Composition and Activity of the Non-canonical Gram-positive SecY2 Complex — SecY2-Asp4-Asp5 Complex of S. gordonii — Supplemental Data 

# Composition and Activity of the Non-canonical Gram-positive SecY2 Complex

## Supplemental Data

- Supplemental data (.pdf, 848 KB) - Supplemental data
