# Supplementary material for: Composition and Activity of the Non-canonical Gram-positive SecY2 Complex
Source: J Biol Chem. 2016 Aug 22;291(41):21474–84. doi: 10.1074/jbc.M116.729806 (PMC5076819; doi:10.1074/jbc.M116.729806)
Supplement: Supplemental Data [file 10.1074_M116.729806_jbc.M116.729806-1.pdf]

SUPPLEMENTAL DATA

Composition and activity of the non-canonical Gram-positive SecY2 complex

Mikaila Bandara[1,2,4], Robin A. Corey[2], Remy Martin[2], J. Mark Skehel[3], Ariel J. Blocker[4,2], Howard F. Jenkinson[1], and Ian Collinson[2]

[1] School of Oral and Dental Sciences, Lower Maudlin Street, Bristol, BS1 2LY, UK
[2] School of Biochemistry, University of Bristol, University Walk, BS8 1TD, UK
[3] Biological Mass Spectrometry and Proteomics, MRC Laboratory of Molecular Biology, Francis Crick Avenue, Cambridge, CB2 0QH, UK
[4] School of Cellular and Molecular Medicine, University of Bristol, University Walk, BS8 1TD, UK

To whom correspondence should be addressed: Ian Collinson, School of Biochemistry, University of Bristol, University Walk, Bristol, BS8 1TD, UK; Telephone: +44 (0) 117 342 4358; Email: ian.collinson@bristol.ac.uk

Running Title: – *The SecY2-Asp4-Asp5 complex of S. gordonii*

Keywords: accessory Sec system, SecY2, *Streptococcus gordonii*, secretion, translocon, membrane, complex, transport, ATPase

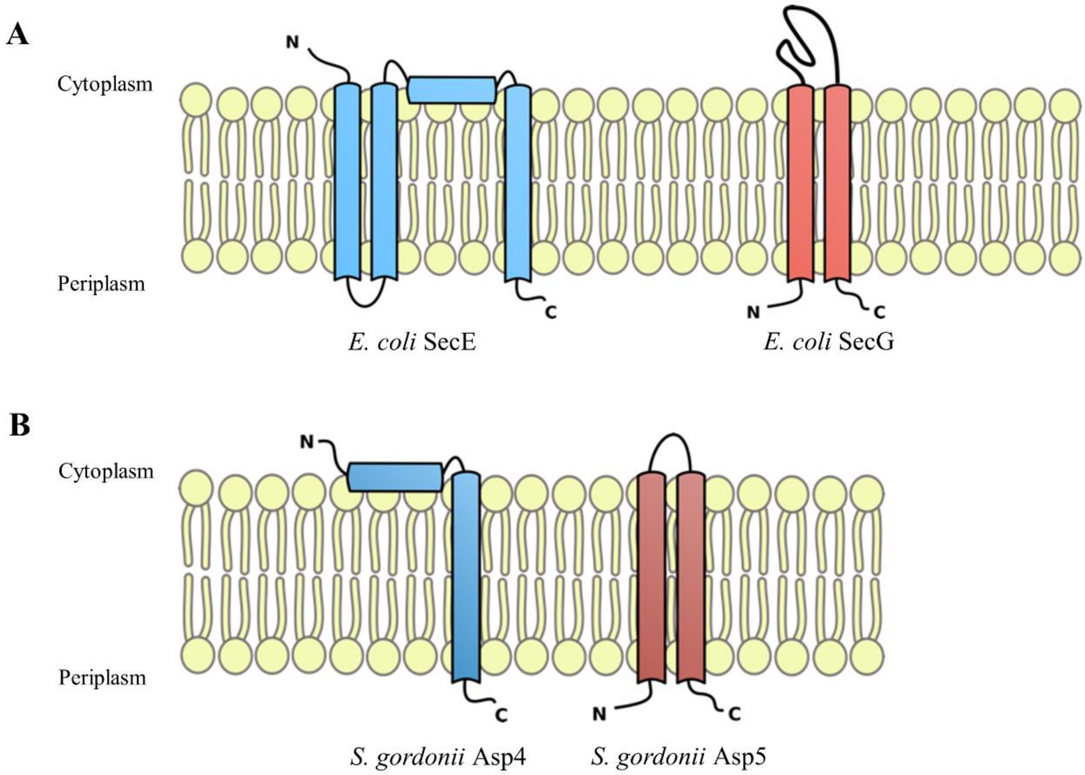

FIGURE S1. Membrane topology map of *E. coli* SecE & SecG, compared to predicted topology of *S. gordonii* Asp4 & Asp5. (A) Schematic of *E.coli* SecE and SecG. SecE is shown in blue and SecG in red. (B) Predicted topology of Asp4 and Asp5 from *S. gordonii*. Asp4 is shown in blue, and Asp5 in red.

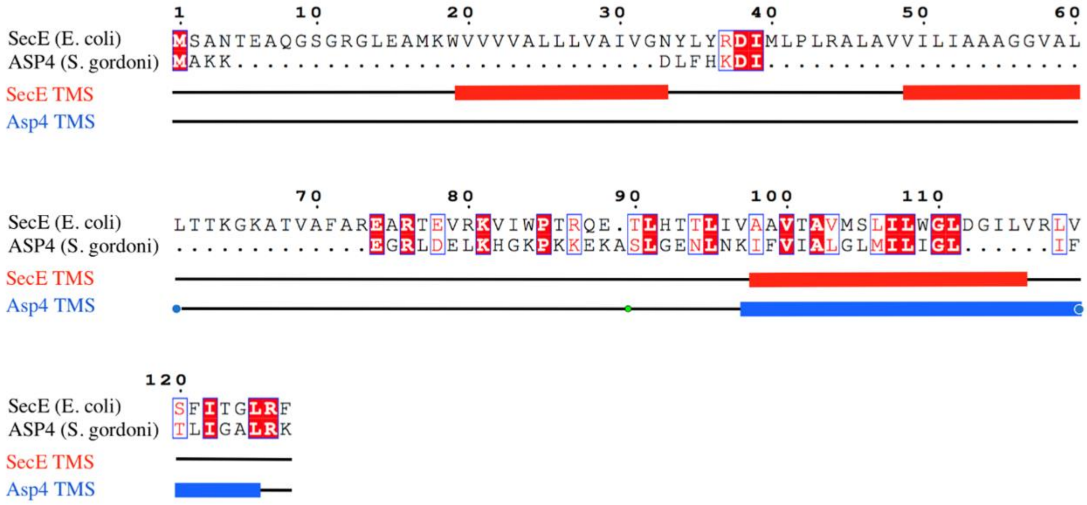

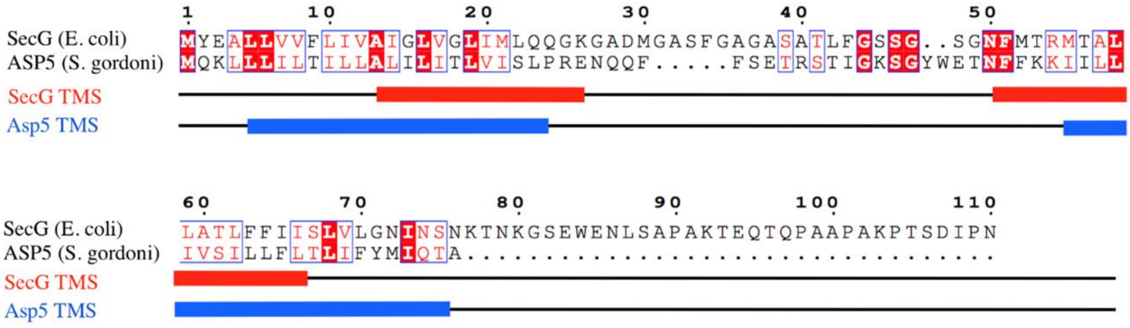

FIGURE S2. Sequence Alignments of SecE & SecG from *E. coli* to Asp4 & Asp5 from *S. gordonii.* Known transmembrane segments (TMS) for *E. coli* are shown as a red bar below the sequence. Predicted TMS for Asp4 and Asp5 are shown as blue bars. TMS predictions were carried out using TMPred (http://www.ch.embnet.org/software/TMPRED_form.html). The global alignment of sequences was performed using MUSCLE (http://www.ebi.ac.uk/Tools/msa/muscle/). Sequence conservation was annotated using ESPript (http://espript.ibcp.fr). Conserved residues are shown as bold white letters on a red background, whilst similar residues are shown as red letters on a white background.

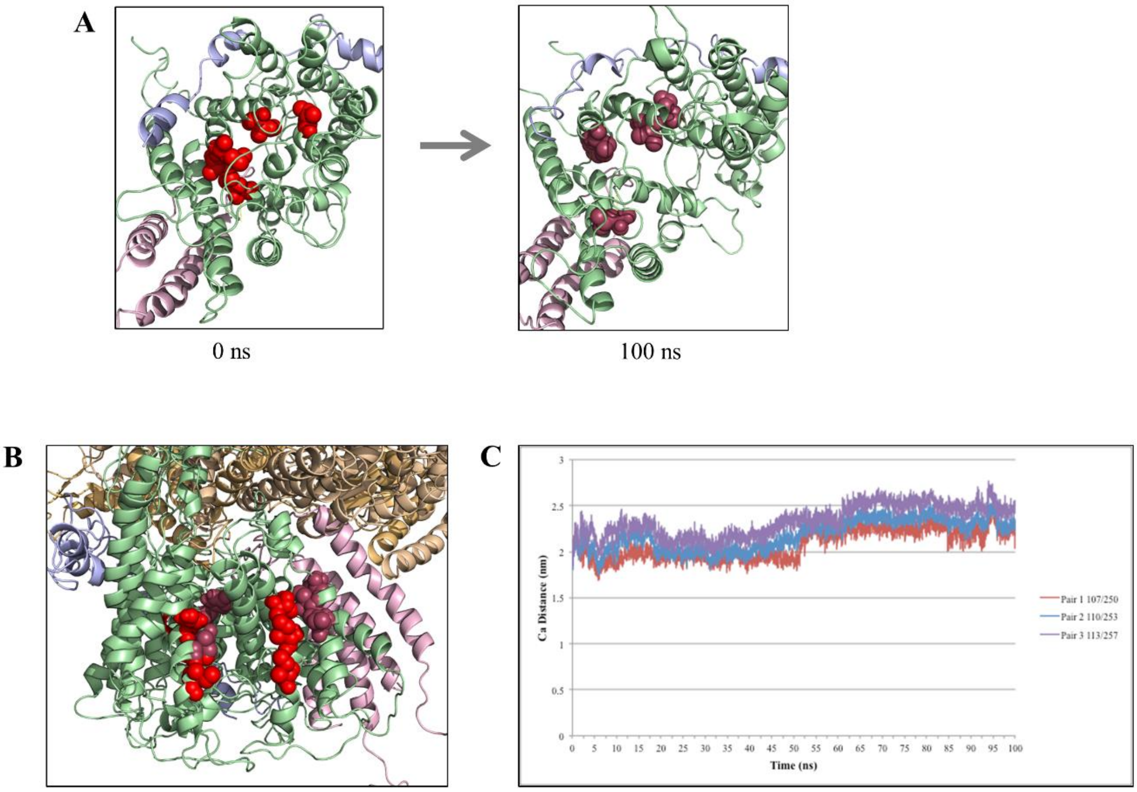

FIGURE S3. Stability of structural features of SecY2 during molecular dynamics simulations. (A) Snapshot of SecY2 viewed from the periplasmic side (SecA2 omitted for clarity) showing conserved pore ring residues highlighted in red (pre simulation) and burgundy (100ns post simulation). Pore ring residues remain remarkably stable, with only L70 moving significantly. (B) SecY2-Asp4-Asp5-SecA2 complex viewed from the side with conserved SecY2 lateral gate residues highlighted in red (pre simulation) and burgundy (100ns post simulation). (C) Graph showing the distance between 3 conserved pairs of lateral gate residues (T107/Y250, I110/T253, I113/I257) over the course of 100ns of MD simulations.

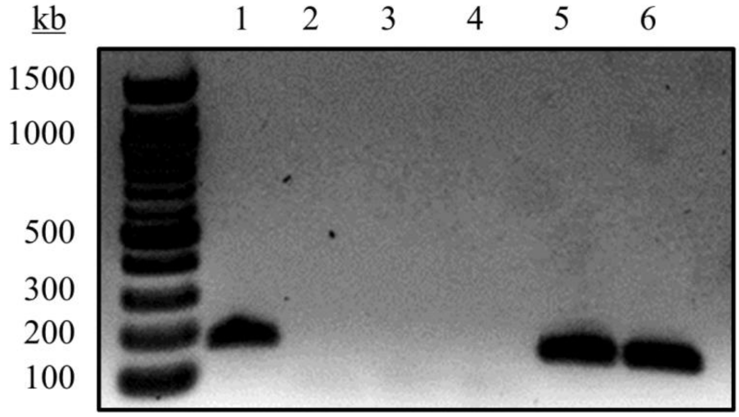

FIGURE S4. Analysis of *asp5* expression. RT-PCR (lanes 1-4) was carried out to check expression of *asp5* in the Δ*asp4* mutant. Primers Asp5RT.F and Asp5RT.R amplified a 190 bp product in the *S. gordonii* wild type (lane 1) and no DNA was detected in the Δ*asp4* mutant (lane 2), confirming no expression of *asp5* in Δ*asp4* mutant. As a negative control (no reverse transcriptase), no DNA was detected for both WT and Δ*asp4* mutant (lane 3 & 4, respectively). As a positive control, primers Asp5RT.F and Asp5RT.R amplified 190 bp products in both chromosomal DNA of WT and Δ*asp4* mutant (lane 5 & 6, respectively).

Table S1. Identification of SecY2 and other proteins by mass spectrometry. Proteins are sorted from highest to lowest emPAI (exponentially modified protein abundance index). SecY2 (accession: A8AWV0) is highlighted in yellow. Other proteins identified are contaminants mainly from *E. coli.*

Acc[a]	Identified proteins	MW[b]	emPAI[c]
A1ABW1	MltA-interacting protein OS=Escherichia coli O1:K1 / APEC GN=yeaF PE=4 SV=1	28	111.57
B1ES24	MltA-interacting protein MipA OS=Escherichia albertii TW07627 GN=mipA PE=4 SV=1	28	13.715
C6EGG3	30S ribosomal protein S5 OS=Escherichia coli (strain B / BL21-DE3) GN=rpsE PE=3 SV=1	18	8.7805
A1ABQ4	Putative uncharacterized protein ydiY OS=Escherichia coli O1:K1 / APEC GN=ydiY PE=4 SV=1	28	4.5111
M8RK73	ProP effector OS=Escherichia coli 2875000 GN=proQ PE=4 SV=1	26	3.2646
C6EAZ8	DnaJ-like protein DjlA OS=Escherichia coli (strain B / BL21-DE3) GN=djlA PE=3 SV=1	31	2.812
A7ZYQ9	Outer membrane protein A OS=Escherichia coli O9:H4 (strain HS) GN=ompA PE=3 SV=1	37	2.0168
A7ZHK0	Protein AmpE OS=Escherichia coli O139:H28 (strain E24377A / ETEC) GN=ampE PE=4 SV=1	32	1.6667
A1AGP2	DNA-binding transcriptional dual regulator OS=Escherichia coli O1:K1 / APEC GN=crp PE=4 SV=1	24	1.5157
B2NCC4	UPF0114 protein YqhA OS=Escherichia coli 53638 GN=yqhA PE=3 SV=1	19	1.2871
B2N9S6	50S ribosomal protein L6 OS=Escherichia coli 53638 GN=rplF PE=3 SV=1	19	1.266
B2N8V8	Rhomboid protease GlpG OS=Escherichia coli 53638 GN=glpG PE=3 SV=1	31	1.2336
A1AG78	Putative export membrane protein OS=Escherichia coli O1:K1 / APEC GN=secG PE=4 SV=1	11	1.2117
A7ZJB9	Protein TolQ OS=Escherichia coli O139:H28 (strain E24377A / ETEC) GN=tolQ PE=3 SV=1	26	1.084
A4W9R6	LPP repeat-containing protein (Precursor) OS=Enterobacter sp. (strain 638) GN=Ent638_1767 PE=3 SV=1	8	0.999
A1A9Z9	Penicillin-binding protein activator LpoB OS=Escherichia coli O1:K1 / APEC GN=lpoB PE=3 SV=1	23	0.99404
A1AA26	High frequency lysogenization protein HflD OS=Escherichia coli O1:K1 / APEC GN=ycfC PE=3 SV=1	23	0.96246
A4WF43	50S ribosomal protein L13 OS=Enterobacter sp. (strain 638) GN=rplM PE=3 SV=1	19	0.91697
A7ZJ72	Negative modulator of initiation of replication OS=Escherichia coli O139:H28 (strain E24377A / ETEC) GN=seqA PE=3 SV=1	20	0.84024
A4MWT3	50S ribosomal protein L5 OS=Haemophilus influenzae 22.1-21 GN=rplE PE=3 SV=1	20	0.84024
B2N2Y7	4-hydroxybenzoate octaprenyltransferase OS=Escherichia coli 53638 GN=ubiA PE=3 SV=1	33	0.78728
B1EQM4	50S ribosomal protein L4 OS=Escherichia albertii TW07627 GN=rplD PE=3 SV=1	22	0.75556
G5Q8M2	50S ribosomal protein L3 OS=Salmonella enterica subsp. enterica serovar Montevideo str. S5-403 GN=LTSEMON_4733 PE=3 SV=1	40	0.74847

A8AWV0	Accessory Sec system protein translocase subunit SecY2 OS=Streptococcus gordonii (strain Challis / ATCC 35105 / CH1 / DL1 / V288) GN=secY2 PE=3 SV=1	46	0.73056
B1ES89	50S ribosomal protein L10 OS=Escherichia albertii TW07627 GN=rplJ PE=3 SV=1	18	0.68724
A7ZK20	Outer membrane protein F OS=Escherichia coli O139:H28 (strain E24377A / ETEC) GN=ompF PE=3 SV=1	39	0.62075
A7ZN24	L-arabinose ABC transporter, permease protein OS=Escherichia coli O139:H28 (strain E24377A / ETEC) GN=araH PE=4 SV=1	34	0.58558
A1AGJ0	Protein translocase subunit SecY OS=Escherichia coli O1:K1 / APEC GN=prlA PE=3 SV=1	49	0.58143
A1ABX5	Conserved predicted lipoprotein OS=Escherichia coli O1:K1 / APEC GN=yeaY PE=4 SV=1		0.56168
A1AIF4	Preprotein translocase SecE subunit OS=Escherichia coli O1:K1 / APEC GN=secE PE=4 SV=1	14	0.55963
A1A7P1	Lipoprotein OS=Escherichia coli O1:K1 / APEC GN=metQ PE=3 SV=1	29	0.53236
B2NBV2	Outer-membrane lipoprotein LolB OS=Escherichia coli 53638 GN=lolB PE=3 SV=1	24	0.48761
A1AHE8	Predicted rhodanese-related sulfurtransferase OS=Escherichia coli O1:K1 / APEC GN=yibN PE=4 SV=1	16	0.48209
A1ABI6	Outer membrane lipoprotein SlyB OS=Escherichia coli O1:K1 / APEC GN=slyB PE=4 SV=1	16	0.47818
A3PKY7	Photosynthetic reaction center, M subunit OS=Rhodobacter sphaeroides (strain ATCC 17029 / ATH 2.4.9) GN=Rsph17029_1899 PE=3 SV=1	35	0.44094
A8ACP2	ATP synthase subunit b OS=Citrobacter koseri (strain ATCC BAA-895 / CDC 4225-83 / SGSC4696) GN=atpF PE=3 SV=1	17	0.42944
B7UFR6	Undecaprenyl-phosphate 4-deoxy-4-formamido-L-arabinose transferase OS=Escherichia coli O127:H6 (strain E2348/69 / EPEC) GN=arnC PE=3 SV=1	36	0.41751
A1AG15	Putative uncharacterized protein OS=Escherichia coli O1:K1 / APEC GN=Ecok1_31110 PE=4 SV=1	18	0.41727
A1A8G3	Putative metal resistance protein OS=Escherichia coli O1:K1 / APEC GN=ybbM PE=4 SV=1	29	0.38053
B7NQF3	Glycosyltransferase WbbA OS=Escherichia coli O7:K1 (strain IAI39 / ExPEC) GN=wbbA PE=4 SV=1	30	0.37567
A1A8Q9	Rod shape-determining protein RodA OS=Escherichia coli O1:K1 / APEC GN=mrdB PE=4 SV=1	40	0.36675
A1AA11	ABC transporter, ATP-binding protein OS=Escherichia coli O1:K1 / APEC GN=ycfV PE=3 SV=1	31	0.35595
B2N133	Electron transport complex, RnfABCDGE type, A subunit OS=Escherichia coli 53638 GN=rnfA PE=3 SV=1	21	0.34605
A1ADR8	Putative permease of transport system for 3 nucleosides OS=Escherichia coli O1:K1 / APEC GN=nupC PE=4 SV=1	43	0.33921
A7ZIG0	Protein translocase subunit SecD OS=Escherichia coli O139:H28 (strain E24377A / ETEC) GN=secD PE=3 SV=1	67	0.33317
A1ABT9	Putative uncharacterized protein ynjF OS=Escherichia coli O1:K1 / APEC GN=ynjF PE=3 SV=1	23	0.31285
A7ZPG1	Long-chain fatty acid transport protein OS=Escherichia coli O139:H28 (strain E24377A / ETEC) GN=EcE24377A_2640 PE=4 SV=1	49	0.2994
A1A9X4	Flagellar L-ring protein OS=Escherichia coli O1:K1 / APEC GN=flgH PE=3 SV=1	25	0.28543

B3X8E3	Cadmium-translocating P-type ATPase OS=Escherichia coli 101-1 GN=cadA PE=3 SV=1	77	0.28419
A7ZI47	Uncharacterized protein OS=Escherichia coli O139:H28 (strain E24377A / ETEC) GN=EcE24377A_0323 PE=4 SV=1	25	0.28132
A6BVS9	30S ribosomal protein S3 OS=Yersinia pestis CA88-4125 GN=rpsC PE=3 SV=1	26	0.27344
A1AGZ9	Cell division protein FtsX OS=Escherichia coli O1:K1 / APEC GN=ftsX PE=4 SV=1	40	0.2714
A1A767	Chaperone protein DnaJ OS=Escherichia coli O1:K1 / APEC GN=dnaJ PE=3 SV=1	42	0.25266
B3HBU8	CDP-diacylglycerol pyrophosphatase OS=Escherichia coli B7A GN=cdh PE=3 SV=1	28	0.24692
A1ABK2	Cyclopropane fatty acyl phospholipid synthase OS=Escherichia coli O1:K1 / APEC GN=cfa PE=4 SV=1	44	0.24221
B1EIL2	Membrane protein insertase YidC OS=Escherichia albertii TW07627 GN=yidC PE=3 SV=1	62	0.23047
A1AEY3	Putative serine transporter OS=Escherichia coli O1:K1 / APEC GN=sdaC PE=4 SV=1	47	0.22541
A0PFL0	Beta-lactamase OS=Acinetobacter baylyi GN=bla PE=4 SV=1	32	0.22083
B3XKQ1	Protease HtpX OS=Escherichia coli 101-1 GN=htpX PE=3 SV=1	32	0.21754
A1AIA8	Essential cell division protein OS=Escherichia coli O1:K1 / APEC GN=ftsN PE=4 SV=1	36	0.19311
A1A879	Protein-export membrane protein SecF OS=Escherichia coli O1:K1 / APEC GN=secF PE=3 SV=1	37	0.18693
B2NCZ7	Phospho-N-acetylmuramoyl-pentapeptide-transferase OS=Escherichia coli 53638 GN=mraY PE=3 SV=1	40	0.17207
F6W7V0	Uncharacterized protein OS=Equus caballus GN=KRT5 PE=3 SV=1	62	0.16575
A8AKU8	Elongation factor Tu OS=Citrobacter koseri (strain ATCC BAA-895 / CDC 4225-83 / SGSC4696) GN=tuf PE=3 SV=1	43	0.1581
B1EIV9	Serine hydroxymethyltransferase OS=Escherichia albertii TW07627 GN=glyA PE=3 SV=1	45	0.15036
A7ZSW8	Gluconate transporter GntU OS=Escherichia coli O139:H28 (strain E24377A / ETEC) GN=gntU PE=4 SV=1	46	0.14659
A1A8A0	Cytochrome o ubiquinol oxidase subunit I OS=Escherichia coli O1:K1 / APEC GN=cyoB PE=3 SV=1	74	0.13762
A1AHV2	Transcription termination factor Rho OS=Escherichia coli O1:K1 / APEC GN=rho PE=3 SV=1	50	0.13664

[a] Protein accession number

[b] Molecular weight (kDa)

[c] Normalized exponentially modified protein abundance index (emPAI)

Table S2. Identification of Asp4, Asp5, and other proteins by mass spectrometry. Proteins are sorted from highest to lowest emPAI (exponentially modified protein abundance index). Asp4 (accession: A8AWV6), Asp (accession: A8AWV7) are highlighted in yellow. SecY2 was also identified (highlighted in yellow), which is most likely a break down product. Other proteins identified are contaminants mainly from *E. coli*.

**Acc**[a]	**Identified proteins**	**MW**[b]	**emPAI**[c]
A1ABI6	Outer membrane lipoprotein SlyB OS=Escherichia coli O1:K1 / APEC GN=slyB PE=4 SV=1	16	9.0862
A1A877	Putative uncharacterized protein yajC OS=Escherichia coli O1:K1 / APEC GN=yajC PE=4 SV=1	12	7.3213
B3X7S8	Putative uncharacterized protein OS=Escherichia coli 101-1 GN=EC1011_4794 PE=4 SV=1	17	3.7381
A8AWV6	Putative uncharacterized protein OS=Streptococcus gordonii (strain Challis / ATCC 35105 / CH1 / DL1 / V288) GN=SGO_0977 PE=4 SV=1	7	3.7162
B1EQN9	50S ribosomal protein L18 OS=Escherichia albertii TW07627 GN=rplR PE=3 SV=1	13	2.3656
B1EKA6	Probable Sec-independent protein translocase protein TatE OS=Escherichia albertii TW07627 GN=tatE PE=3 SV=1	7	1.8646
A1AHE8	Predicted rhodanese-related sulfurtransferase OS=Escherichia coli O1:K1 / APEC GN=yibN PE=4 SV=1	16	1.7949
B3APG9	30S ribosomal protein S10 OS=Escherichia coli O157:H7 str. EC4486 GN=rpsJ PE=3 SV=1	12	1.7266
B1EQM8	50S ribosomal protein L22 OS=Escherichia albertii TW07627 GN=rplV PE=3 SV=1	12	1.644
B2NCC4	UPF0114 protein YqhA OS=Escherichia coli 53638 GN=yqhA PE=3 SV=1	19	1.4075
A7ZRY7	Uncharacterized protein OS=Escherichia coli O139:H28 (strain E24377A / ETEC) GN=EcE24377A_3571 PE=4 SV=1	11	1.0714
A1AG78	Putative export membrane protein OS=Escherichia coli O1:K1 / APEC GN=secG PE=4 SV=1	11	1.0463
B2N0S4	Cell division protein FtsB OS=Escherichia coli 53638 GN=ftsB PE=3 SV=1	12	1.0108
A1ABS0	Osmotically inducible lipoprotein E OS=Escherichia coli O1:K1 / APEC GN=osmE PE=4 SV=1	12	0.97766
A7ZSI7	30S ribosomal protein S13 OS=Escherichia coli O139:H28 (strain E24377A / ETEC) GN=rpsM PE=3 SV=1	13	0.88103
A1AIF4	Preprotein translocase SecE subunit OS=Escherichia coli O1:K1 / APEC GN=secE PE=4 SV=1	14	0.83946
A1A7C8	Cell division protein FtsL OS=Escherichia coli O1:K1 / APEC GN=ftsL PE=3 SV=1	14	0.83946
B1EQN7	30S ribosomal protein S8 OS=Escherichia albertii TW07627 GN=rpsH PE=3 SV=1	14	0.80889
B2N9U1	Large-conductance mechanosensitive channel OS=Escherichia coli 53638 GN=mscL PE=3 SV=1	15	0.75391
B1EQP2	50S ribosomal protein L15 OS=Escherichia albertii TW07627 GN=rplO PE=3 SV=1	15	0.75391
A7ZV45	Uncharacterized protein OS=Escherichia coli O139:H28 (strain E24377A / ETEC) GN=EcE24377A_4735 PE=4 SV=1	7	0.73417
B1EQP0	30S ribosomal protein S5 OS=Escherichia albertii TW07627 GN=rpsE PE=3 SV=1	18	0.63038

A8AWV7	Putative uncharacterized protein OS=Streptococcus gordonii (strain Challis / ATCC 35105 / CH1 / DL1 / V288) GN=SGO_0978 PE=4 SV=1	8	0.62081
H6P243	UPF0114 protein YqhA OS=Salmonella enterica subsp. enterica serovar Typhi str. P-stx-12 GN=yqhA PE=3 SV=1	19	0.59232
L7ZKJ3	UPF0114 protein yqhA OS=Serratia marcescens WW4 GN=yqhA PE=3 SV=1	19	0.59232
A1AH39	Outer membrane protein induced after carbon starvation OS=Escherichia coli O1:K1 / APEC GN=slp PE=4 SV=1	22	0.48479
A1A8Y5	Putative uncharacterized protein OS=Escherichia coli O1:K1 / APEC GN=Ecok1_06310 PE=4 SV=1	11	0.47393
A1AEE0	30S ribosomal protein S16 OS=Escherichia coli O1:K1 / APEC GN=rpsP PE=3 SV=1	11	0.4497
A7ZS71	Preprotein translocase, SecG subunit OS=Escherichia coli O139:H28 (strain E24377A / ETEC) GN=secG PE=4 SV=1	11	0.4497
E0R702	Preprotein translocase subunit SecG OS=Escherichia coli NC101 GN=secG PE=4 SV=1	11	0.4497
B1EFK1	50S ribosomal protein L21 OS=Escherichia albertii TW07627 GN=rplU PE=3 SV=1	12	0.44515
B2N1N3	50S ribosomal protein L19 OS=Escherichia coli 53638 GN=rplS PE=3 SV=1	13	0.38986
B2N210	Fumarate reductase subunit D OS=Escherichia coli 53638 GN=frdD PE=3 SV=1	13	0.38986
A7ZUR4	Diacylglycerol kinase OS=Escherichia coli O139:H28 (strain E24377A / ETEC) GN=dgkA PE=4 SV=1	13	0.38643
A1ABI8	Putative uncharacterized protein ydhJ OS=Escherichia coli O1:K1 / APEC GN=ydhJ PE=4 SV=1	27	0.385
B2N9S1	50S ribosomal protein L14 OS=Escherichia coli 53638 GN=rplN PE=3 SV=1	14	0.37649
A7MJC3	30S ribosomal protein S9 OS=Cronobacter sakazakii (strain ATCC BAA-894) GN=rpsI PE=3 SV=1	15	0.33612
B2VEN6	Outer membrane lipoprotein OS=Erwinia tasmaniensis (strain DSM 17950 / Et1/99) GN=slyB PE=4 SV=1	15	0.32857
C6DIA4	Rhodanese domain protein OS=Pectobacterium carotovorum subsp. carotovorum (strain PC1) GN=PC1_4083 PE=4 SV=1	16	0.32372
A6BXN4	50S ribosomal protein L13 OS=Yersinia pestis CA88-4125 GN=rplM PE=3 SV=1	16	0.3167
A7ZYG9	Putative lipoprotein OS=Escherichia coli O9:H4 (strain HS) GN=EcHS_A0969 PE=4 SV=1	19	0.26501
B1EQN5	50S ribosomal protein L5 OS=Escherichia albertii TW07627 GN=rplE PE=3 SV=1	20	0.24707
A1ADR8	Putative permease of transport system for 3 nucleosides OS=Escherichia coli O1:K1 / APEC GN=nupC PE=4 SV=1	43	0.23587
A9MSZ7	50S ribosomal protein L4 OS=Salmonella paratyphi B (strain ATCC BAA-1250 / SPB7) GN=rplD PE=3 SV=1	22	0.23141
A7ZJB9	Protein TolQ OS=Escherichia coli O139:H28 (strain E24377A / ETEC) GN=tolQ PE=3 SV=1	26	0.19529
D2AXY4	Uncharacterized protein OS=Streptosporangium roseum (strain ATCC 12428 / DSM 43021 / JCM 3005 / NI 9100) GN=Sros_2172 PE=4 SV=1	27	0.18695
A1ABW1	MltA-interacting protein OS=Escherichia coli O1:K1 / APEC GN=yeaF PE=4 SV=1	28	0.17856
A6FAF7	ABC transporter, ATP-binding protein OS=Moritella sp. PE36 GN=PE36_19220 PE=4 SV=1	59	0.17263

A4CRU4	UDP-N-acetylglucosamine 2-epimerase OS=Synechococcus sp. (strain WH7805) GN=WH7805_13713 PE=3 SV=1	40	0.12255
A8AWV0	Accessory Sec system protein translocase subunit SecY2 OS=Streptococcus gordonii (strain Challis / ATCC 35105 / CH1 / DL1 / V288) GN=secY2 PE=3 SV=1	46	0.10644
A7ZIG0	Protein translocase subunit SecD OS=Escherichia coli O139:H28 (strain E24377A / ETEC) GN=secD PE=3 SV=1	67	0.073642
A1A8C9	Acridine efflux pump OS=Escherichia coli O1:K1 / APEC GN=acrB PE=4 SV=1	114	0.043005

[a] Protein accession number

[b] Molecular weight (kDa)

[c] Normalized exponentially modified protein abundance index (emPAI)
